# Supplementary material for: Determining the Virulence Properties of Escherichia coli ST131 Containing Bacteriocin-Encoding Plasmids Using Short- and Long-Read Sequencing and Comparing Them with Those of Other E. coli Lineages
Source: Microorganisms. 2019 Nov 6;7(11):534. doi: 10.3390/microorganisms7110534 (PMC6920910; doi:10.3390/microorganisms7110534)
Supplement: Supplementary file 1 [file microorganisms-07-00534-s001.pdf]

Supplementary Table

Table S1. Bacteriocin genes identified among ExPEC isolates.

	ID	ST	Phylogenetic group	Type of bacteriocin
Bacteriocin-producer isolates	9307	ST91	B2	Colicin_A, Colicin_Ib, Colicin_E9, Colicin_Y, Microcin_B17
	5332	ST131	B2	Colicin_E1, Colicin_Ia, Pyocin_S
	5848	ST131	B2	Microcin_V, Colicin_Ia, Pyocin_S, Colicin_E1
	5038	ST58	B1	Microcin_V, Colicin_E1, Colicin_Ia
	5306	ST641	B1	Colicin_Ia, Microcin_V, Colicin-A, Colicin-M
	1825	ST93	A	Pyocin_S, Colicin-M, Colicin_Ia
	3921	ST101	B1	Colicin_Ia
	7167	ST1431	B1	Microcin_V, Colicin_Ia
	6419	ST676	B2	Pyocin_S, Colicin_Ia, Colicin-M
	9097	ST95	B2	Pyocin_S, Colicin_Ia
	6632D	ST453	B1	Colicin_Ia
	7500	ST744	A	Pyocin_S, Colicin_Ia
	6492	ST12	B2	Colicin_E9, Colicin_Ia
	7078	ST131	B2	Colicin_E1, Colicin_Ia, Pyocin_S
non-bacteriocin producer isolates	7018	ST131	B2	Pyocin_S
	7104	ST131	B2	Pyocin_S, Colicin_E1
	9260	ST131	B2	Pyocin_S
	9581A	ST131	B2	Pyocin_S
	7974	ST131	B2	Pyocin_S
	5976	ST131	B2	Pyocin_S, Colicin_E1
	8565	ST131	B2	Pyocin_S
	9893	ST131	B2	Pyocin_S, Colicin_E1
	3218	ST131	B2	Pyocin_S, Colicin_E1
	6202	ST131	B2	Pyocin_S
	1294D	ST131	B2	Pyocin_S
	3528	ST131	B2	Pyocin_S
	4233	ST131	B2	Pyocin_S
	5770D	ST131	B2	Pyocin_S
	1710D	ST131	B2	Pyocin_S
	9533D	ST131	B2	Pyocin_S
	2724	ST131	B2	Pyocin_S
	2206	ST131	B2	Pyocin_S
	5420	ST131	B2	Pyocin_S
	6638	ST131	B2	Pyocin_S
	2102	ST131	B2	Pyocin_S
	2478	ST131	B2	Pyocin_S
	4006	ST131	B2	Pyocin_S
	7348	ST73	B2	Colicin-10, Colicin_E9

	2723A	ST73	B2	Colicin-E1, Colicin_E9
	3052	ST73	B2	Colicin_E9
	9492	ST73	B2	Colicin_E9, Pyocin_S
	6077	ST10	A	Microcin_B17
	8874	ST10	A	Microcin_V, Colicin-A, Colicin-M
	5217	ST10	A	Colicin-M, Colicin-A
	8200	ST10	A	Colicin_E1
	3188	ST10	A	None
	9733D	ST10	A	None
	0015D	ST10	A	None
	2986	ST648	B2	None
	1843	ST648	B2	None
	7002	ST648	B2	None
	6022	ST648	B2	None
	2993	ST648	B2	None
	0107D	ST648	B2	None
	605	ST69	D	None
	2441	ST69	D	None
	108	ST69	D	None
	9715	ST69	D	None
	666	ST69	D	None
	4953	ST69	D	None
	2445A	ST69	D	None
	7719	ST69	D	None
	864	ST69	D	None
	2877	ST405	D	None
	6161	ST405	D	None
	9602	ST405	D	None
	6050	ST405	D	None

Supplementary Figures

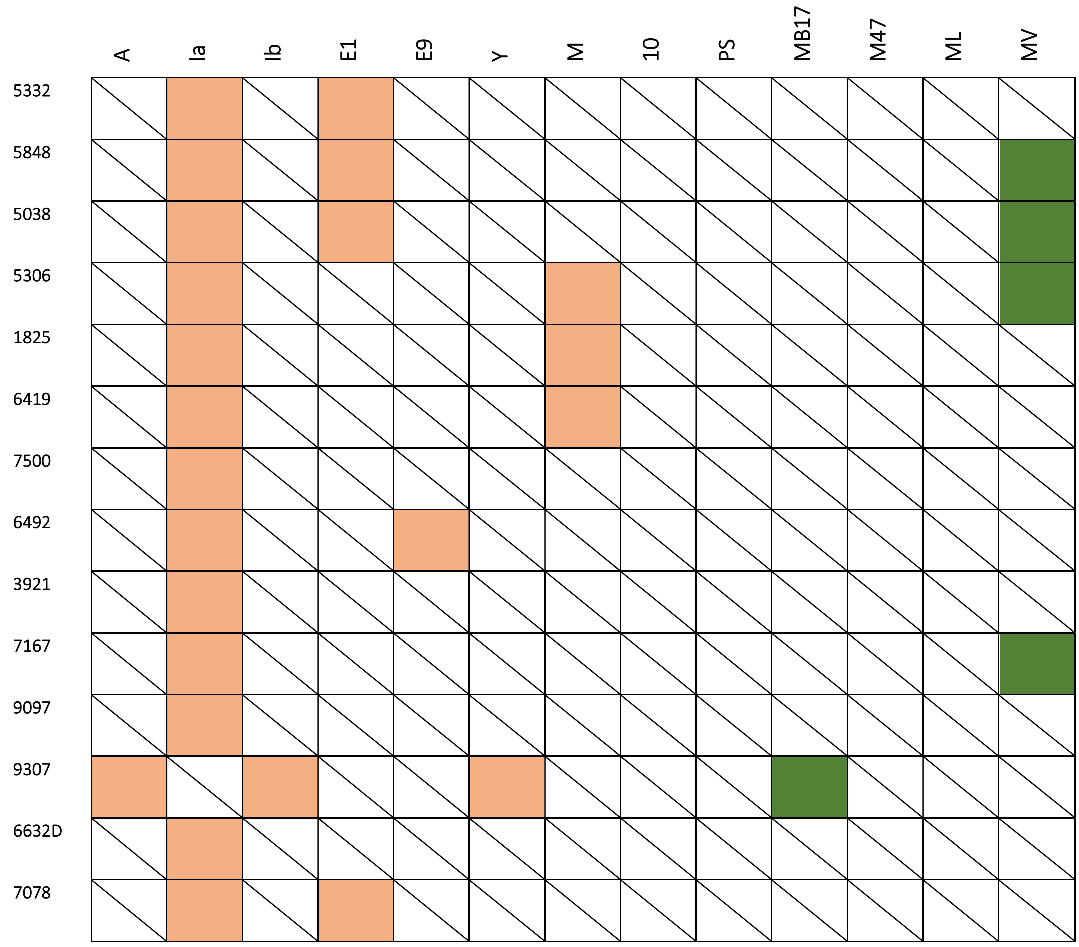

Figure S1. Bacteriocin genes identified among the bacteriocin producer-isolates. The orange squares indicate the presence of colicin genes in the isolates, while the green squares indicate the presence of microcin genes in these isolates and the diagonal

A

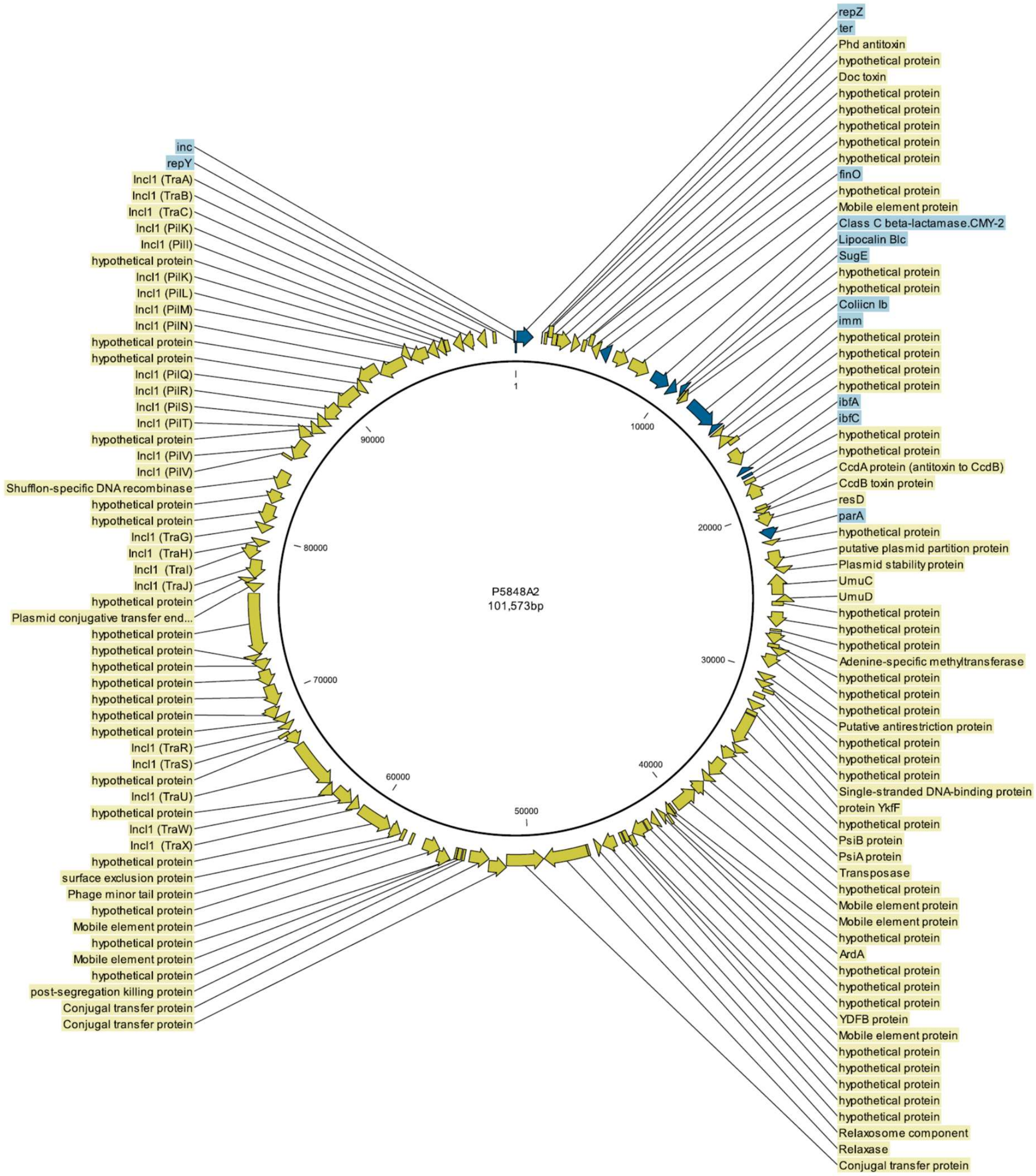

inc
repY
IncI1 (TraA)
IncI1 (TraB)
IncI1 (TraC)
IncI1 (PilK)
IncI1 (PilI)
hypothetical protein
IncI1 (PilK)
IncI1 (PilL)
IncI1 (PilM)
IncI1 (PilN)
hypothetical protein
hypothetical protein
IncI1 (PilQ)
IncI1 (PilR)
IncI1 (PilS)
IncI1 (PilT)
hypothetical protein
IncI1 (PilV)
IncI1 (PilV)
Shufflon-specific DNA recombinase
hypothetical protein
hypothetical protein
IncI1 (TraG)
IncI1 (TraH)
IncI1 (TraI)
IncI1 (TraJ)
hypothetical protein
Plasmid conjugative transfer end...
hypothetical protein
hypothetical protein
hypothetical protein
hypothetical protein
hypothetical protein
hypothetical protein
hypothetical protein
IncI1 (TraR)
IncI1 (TraS)
hypothetical protein
IncI1 (TraU)
hypothetical protein
IncI1 (TraW)
IncI1 (TraX)
hypothetical protein
surface exclusion protein
Phage minor tail protein
hypothetical protein
Mobile element protein
hypothetical protein
Mobile element protein
hypothetical protein
post-segregation killing protein
Conjugal transfer protein
Conjugal transfer protein
repZ
ter
Phd antitoxin
hypothetical protein
Doc toxin
hypothetical protein
hypothetical protein
hypothetical protein
hypothetical protein
hypothetical protein
finO
hypothetical protein
Mobile element protein
Class C beta-lactamase.CMY-2
Lipocalin Blc
SugE
hypothetical protein
hypothetical protein
Coliicn Ib
imm
hypothetical protein
hypothetical protein
hypothetical protein
hypothetical protein
ibfA
ibfC
hypothetical protein
hypothetical protein
CcdA protein (antitoxin to CcdB)
CcdB toxin protein
resD
parA
hypothetical protein
putative plasmid partition protein
Plasmid stability protein
UmuC
UmuD
hypothetical protein
hypothetical protein
hypothetical protein
Adenine-specific methyltransferase
hypothetical protein
hypothetical protein
hypothetical protein
Putative antirestriction protein
hypothetical protein
hypothetical protein
hypothetical protein
Single-stranded DNA-binding protein
protein YkfF
hypothetical protein
PsiB protein
PsiA protein
Transposase
hypothetical protein
Mobile element protein
Mobile element protein
hypothetical protein
ArdA
hypothetical protein
hypothetical protein
hypothetical protein
YDFB protein
Mobile element protein
hypothetical protein
hypothetical protein
hypothetical protein
hypothetical protein
hypothetical protein
Relaxosome component
Relaxase
Conjugal transfer protein
P5848A2
101,573bp
1
10000
20000
30000
40000
50000
60000
70000
80000
90000

B

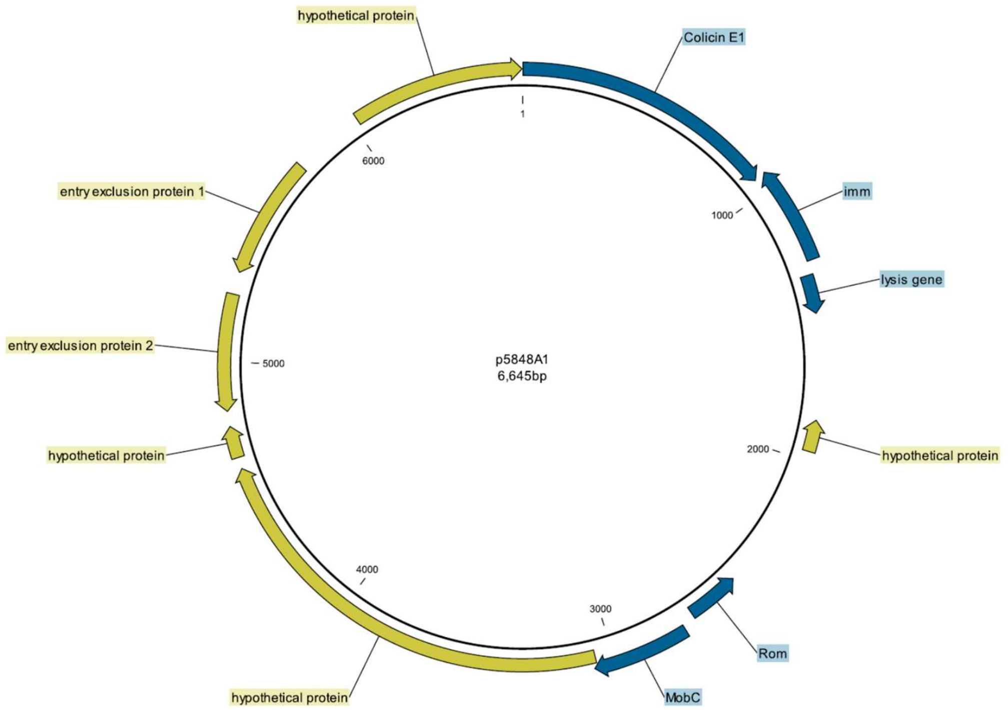
hypothetical protein
Colicin E1
1
6000
entry exclusion protein 1
1000
imm
lysis gene
entry exclusion protein 2
5000
p5848A1
6,645bp
2000
hypothetical protein
hypothetical protein
4000
3000
Rom
MobC
hypothetical protein

C

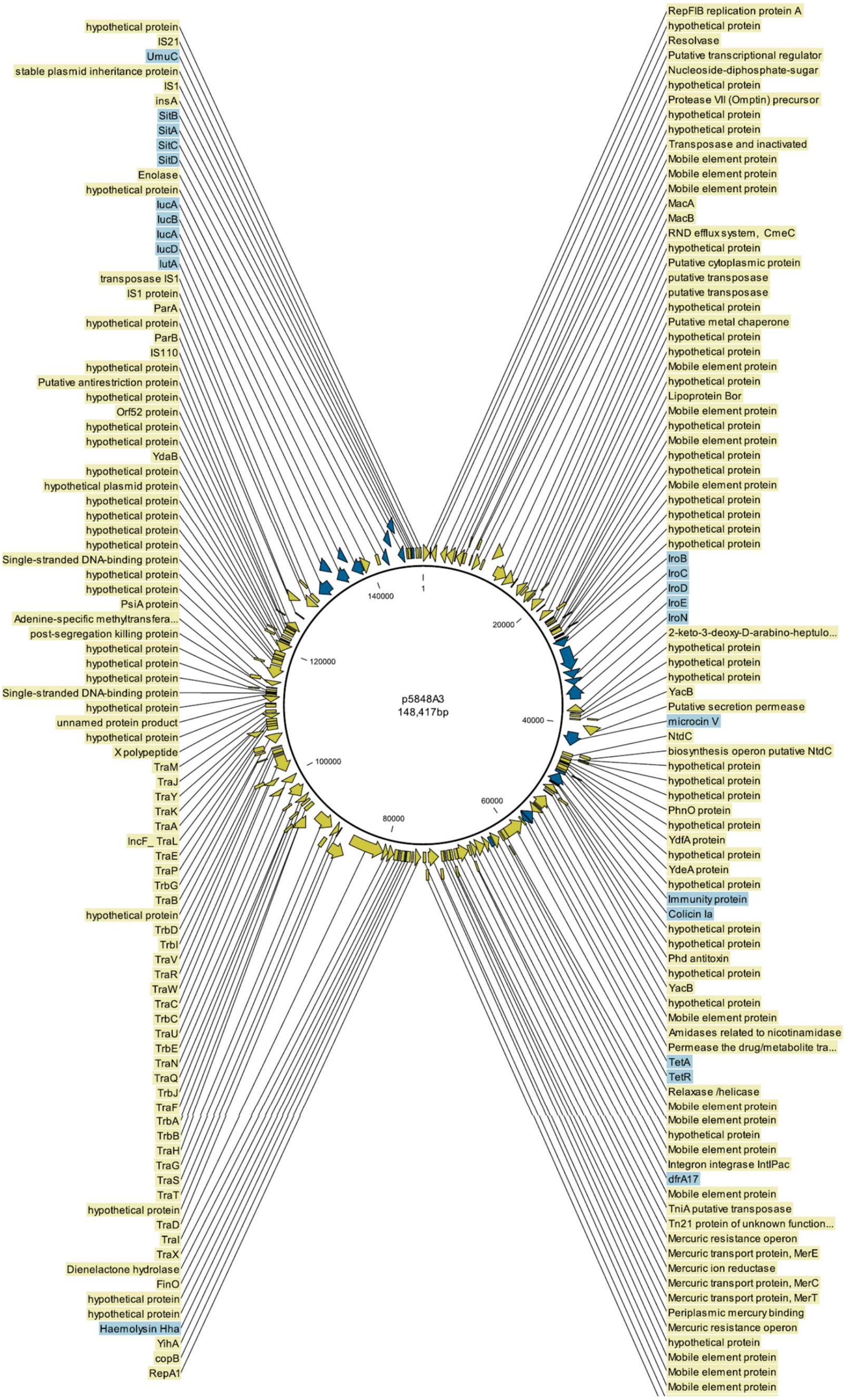
hypothetical protein
IS21
UmuC
stable plasmid inheritance protein
IS1
insA
SitB
SitA
SitC
SitD
Enolase
hypothetical protein
IucA
IucB
IucA
IucD
IutA
transposase IS1
IS1 protein
ParA
hypothetical protein
ParB
IS110
hypothetical protein
Putative antirestriction protein
hypothetical protein
Orf52 protein
hypothetical protein
hypothetical protein
YdaB
hypothetical protein
hypothetical plasmid protein
hypothetical protein
hypothetical protein
hypothetical protein
hypothetical protein
Single-stranded DNA-binding protein
hypothetical protein
hypothetical protein
PsiA protein
Adenine-specific methyltransfera...
post-segregation killing protein
hypothetical protein
hypothetical protein
hypothetical protein
Single-stranded DNA-binding protein
hypothetical protein
unnamed protein product
hypothetical protein
X polypeptide
TraM
TraJ
TraY
TraK
TraA
IncF_ TraL
TraE
TraP
TrbG
TraB
hypothetical protein
TrbD
TrbI
TraV
TraR
TraW
TraC
TrbC
TraU
TrbE
TraN
TraQ
TrbJ
TraF
TrbA
TrbB
TraH
TraG
TraS
TraT
hypothetical protein
TraD
TraI
TraX
Dienelactone hydrolase
FinO
hypothetical protein
hypothetical protein
Haemolysin Hha
YihA
copB
RepA1
RepFIB replication protein A
hypothetical protein
Resolvase
Putative transcriptional regulator
Nucleoside-diphosphate-sugar
hypothetical protein
Protease VII (Omptin) precursor
hypothetical protein
hypothetical protein
Transposase and inactivated
Mobile element protein
Mobile element protein
Mobile element protein
MacA
MacB
RND efflux system, CmeC
hypothetical protein
Putative cytoplasmic protein
putative transposase
putative transposase
hypothetical protein
Putative metal chaperone
hypothetical protein
hypothetical protein
Mobile element protein
hypothetical protein
Lipoprotein Bor
Mobile element protein
hypothetical protein
Mobile element protein
hypothetical protein
hypothetical protein
Mobile element protein
hypothetical protein
hypothetical protein
hypothetical protein
hypothetical protein
IroB
IroC
IroD
IroE
IroN
2-keto-3-deoxy-D-arabino-heptulo...
hypothetical protein
hypothetical protein
hypothetical protein
YacB
Putative secretion permease
microcin V
NtdC
biosynthesis operon putative NtdC
hypothetical protein
hypothetical protein
hypothetical protein
PhnO protein
hypothetical protein
YdfA protein
hypothetical protein
YdeA protein
hypothetical protein
Immunity protein
Colicin Ia
hypothetical protein
hypothetical protein
Phd antitoxin
hypothetical protein
YacB
hypothetical protein
Mobile element protein
Amidases related to nicotinamidase
Permease the drug/metabolite tra...
TetA
TetR
Relaxase /helicase
Mobile element protein
Mobile element protein
hypothetical protein
Mobile element protein
Integron integrase IntIPac
dfrA17
Mobile element protein
TniA putative transposase
Tn21 protein of unknown function...
Mercuric resistance operon
Mercuric transport protein, MerE
Mercuric ion reductase
Mercuric transport protein, MerC
Mercuric transport protein, MerT
Periplasmic mercury binding
Mercuric resistance operon
hypothetical protein
Mobile element protein
Mobile element protein
Mobile element protein
p5848A3
148,417bp
1
20000
40000
60000
80000
100000
120000
140000

D

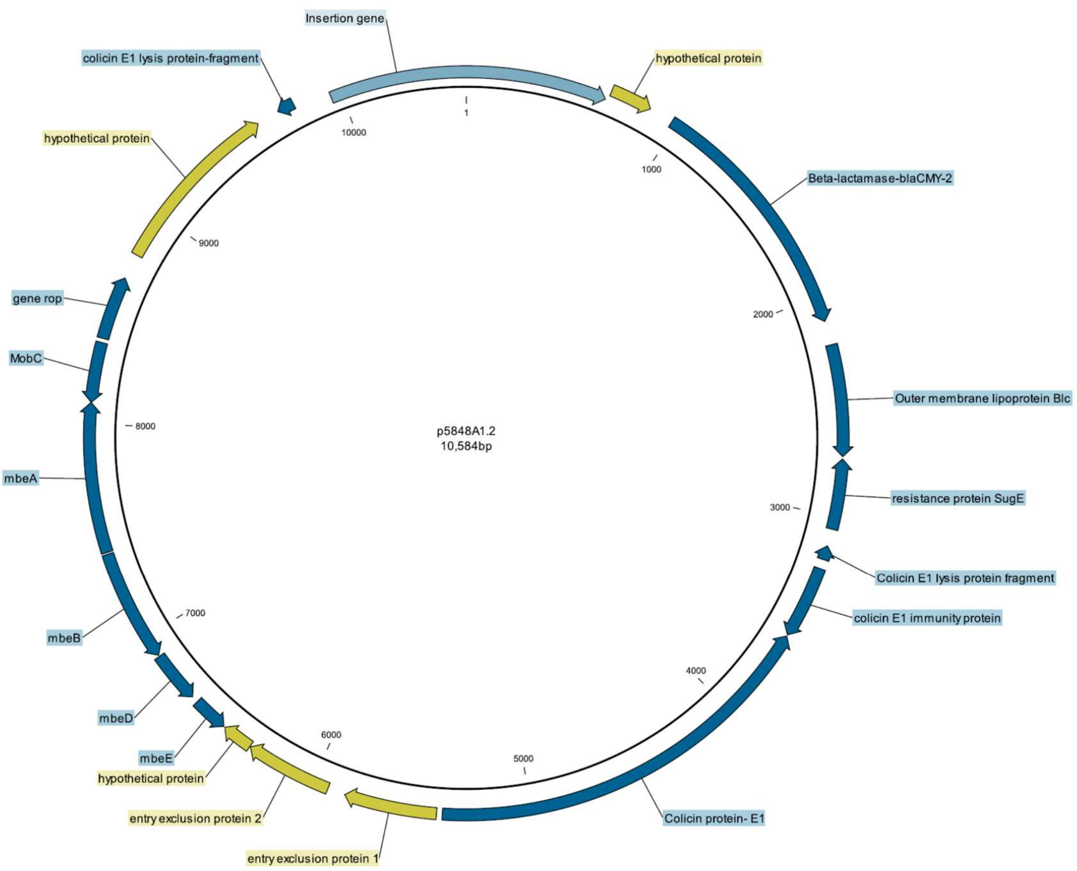

Figure S2. Plasmids Sequences **(A)** complete annotated sequence of plasmid p5848A2, highlighted in blue are the antibiotic resistance gene beta-lactamase *bla*$_{CMY-2}$, the *blc* and *sugE* genes, the colicin *Ib* gene, and the *imm* gene encoding the immunity protein; **(B)** Complete sequence of the plasmid p5848A1 indicating the E1 colicin gene, the *imm* gene encoding the immunity protein and the gene encoding the colicin E1 precursor lysis protein; **(C)** complete annotated sequence of plasmid p5848A3**,** highlighted in blue are the antibiotic resistance and virulence genes, and the colicin Ia and microcin V genes; **(D)** the complete sequence of plasmid p5848A1.2, containing the antibiotic resistance gene beta-lactamase *bla*$_{CMY-2}$, the *blc* and *sugE* genes, and the colicin *Ib* gene.

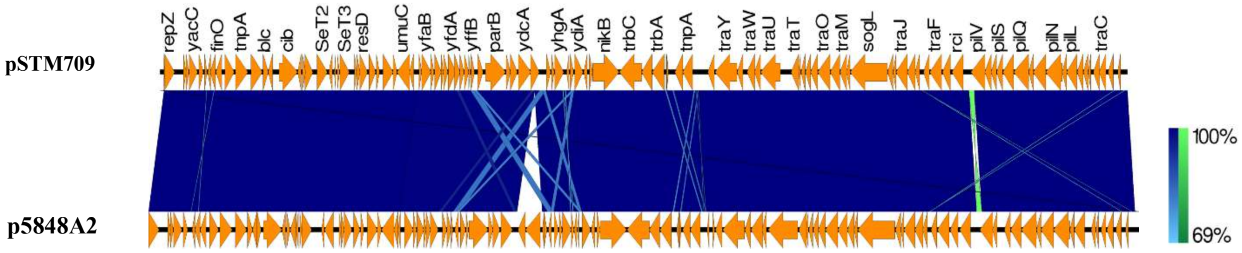

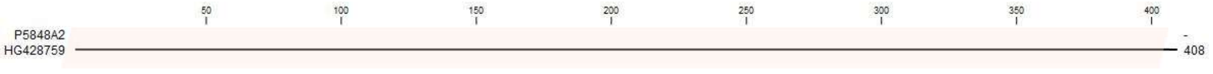

Region 1 (nt 2-408)

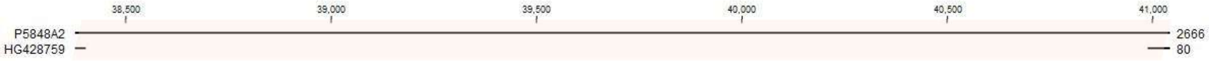

Region 2 (nt 38446-41078)

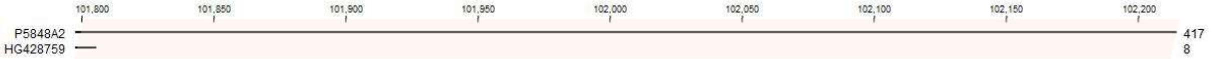

Region 3 (nt 101808-101573)

Figure S3. Alignment of plasmids p5848A2 and pSTM709. Alignment of plasmid p5848A2 present in isolates 5332 and 5848 and pSTM709 (HG428759) present in *Salmonella enterica* isolated in Uruguay. The orange trapeziums in the lower part of the figure represent the regions present in plasmid p5848A2 but absent in the *Salmonella enterica* plasmid pSTM709.

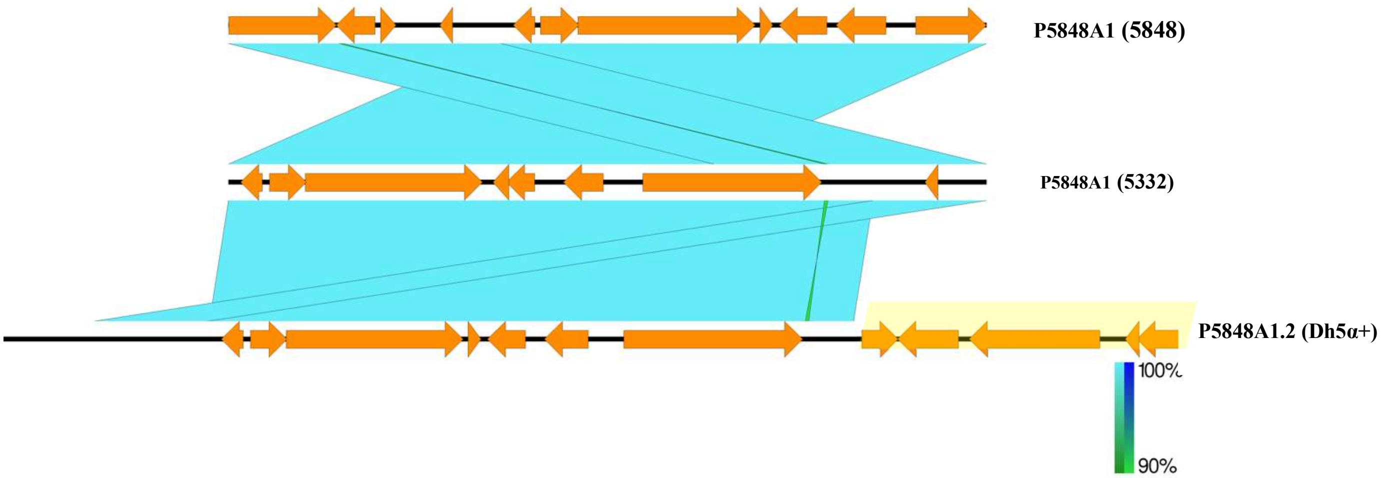

Figure S4. Comparison of plasmids p5848A1 and p5848A2.1. Alignment of plasmids p5848A1 present in the isolates 5848 and 5332 and the p5848A1.2 plasmid present in the Dh5α+ mutant after recombination. The fragment containing the resistance gene and the flanked genes that recombined with p5848A1 plasmid is highlighted in yellow.
